# Urogenital schistosomiasis elimination in Zanzibar: accuracy of urine filtration and haematuria reagent strips for diagnosing light intensity *Schistosoma haematobium* infections

**DOI:** 10.1186/s13071-018-3136-6

**Published:** 2018-10-23

**Authors:** Stefanie Knopp, Shaali M Ame, Jan Hattendorf, Said M Ali, Iddi S Khamis, Faki Bakar, Mwanaidi A Khamis, Bobbie Person, Fatma Kabole, David Rollinson

**Affiliations:** 1 Swiss Tropical and Public Health Institute, Socinstrasse 57, 4002 Basel, Switzerland; 2 University of Basel, Petersplatz 1, 4001 Basel, Switzerland; 3 Wolfson Wellcome Biomedical Laboratories, Department of Life Sciences, Natural History Museum, Cromwell Road, London, SW7 5BD UK; 4 Public Health Laboratory Ivo-de Carneri, P.O. Box 122, Chake-Chake, Pemba United Republic of Tanzania; 5 Neglected Diseases Programme, Ministry of Health, P.O. Box 236, Unguja, United Republic of Tanzania; 6 Schistosomiasis Consortium for Operational Research and Evaluation, 145 Coverdell Center, The University of Georgia, 500 D.W. Brooks Drive, Athens, Georgia 30602 USA

**Keywords:** Control, Diagnosis, Elimination, Macrohaematuria, Microhaematuria, Reagent strip, *Schistosoma haematobium*, Surveillance, Urine filtration, Urogenital schistosomiasis, Zanzibar

## Abstract

**Background:**

Urine filtration and microhaematuria reagent strips are basic standard diagnostic methods to detect urogenital schistosomiasis. We assessed their accuracy for the diagnosis of light intensity infections with *Schistosoma haematobium* as they occur in individuals living in Zanzibar, an area targeted for interruption of transmission.

**Methods:**

Urine samples were collected from children and adults in surveys conducted annually in Zanzibar from 2013 through 2016 and examined with the urine filtration method to count *S. haematobium* eggs and with the reagent strip test (Hemastix) to detect microhaematuria as a proxy for infection. Ten percent of the urine filtration slides were read twice. Sensitivity was calculated for reagent strips, stratified by egg counts reflecting light intensity sub-groups, and kappa statistics for the agreement of urine filtration readings.

**Results:**

Among the 39,207 and 18,155 urine samples examined from children and adults, respectively, 5.4% and 2.7% were *S. haematobium* egg-positive. A third (34.7%) and almost half (46.7%) of the egg-positive samples from children and adults, respectively, had ultra-low counts defined as 1–5 eggs per 10 ml urine. Sensitivity of the reagent strips increased significantly for each unit log10 egg count per 10 ml urine in children (odds ratio, OR: 4.7; 95% confidence interval, CI: 4.0–5.7; *P* < 0.0001) and adults (OR: 2.6; 95% CI: 1.9–3.7, *P* < 0.0001). Sensitivity for diagnosing ultra-light intensity infections was very low in children (50.1%; 95% CI: 46.5–53.8%) and adults (58.7%; 95% CI: 51.9–65.2%). Among the 4477 and 1566 urine filtration slides read twice from children and adults, most were correctly identified as negative or positive (kappa = 0.84 for children and kappa = 0.81 for adults). However, 294 and 75 slides had discrepant results and were positive in only one of the two readings. The majority of these discrepant slides (76.9% of children and 84.0% of adults) had counts of 1–5 eggs per 10 ml urine.

**Conclusions:**

We found that many individuals infected with *S. haematobium* in Zanzibar excrete > 5 eggs per 10 ml urine. These ultra-light infections impose a major challenge for accurate diagnosis. Next-generation diagnostic tools to be used in settings where interruption of transmission is the goal should reliably detect infections with ≤ 5 eggs per 10 ml urine.

**Trial Registration:**

ISRCTN, ISRCTN48837681. Registered 05 September 2012 - Retrospectively registered.

**Electronic supplementary material:**

The online version of this article (10.1186/s13071-018-3136-6) contains supplementary material, which is available to authorized users.

## Background

Urogenital schistosomiasis, caused by the blood fluke *Schistosoma haematobium*, is a common neglected tropical disease (NTD) in many countries of sub-Saharan Africa and the Middle East [1, 2]. In 2012, the World Health Organization (WHO) encouraged endemic countries to increase the coverage of preventive chemotherapy programmes for the control of morbidity due to schistosomiasis and to initiate elimination campaigns where appropriate, through strengthened health systems, intensified treatment, provision of water and sanitation, addition of health education for behaviour change and snail control to the programmes [3, 4]. As a reaction, over the past years, efforts for control and elimination of schistosomiasis have substantially increased.

The typical sign for urogenital schistosomiasis is the presence of blood in urine [5]. High-risk communities can be identified by using a simple questionnaire asking for visible blood (macrohaematuria) in urine [6, 7]. Another recommended proxy for a *S. haematobium* infection is the detection of microhaematuria using reagent strips [8–10]. The standard method for urogenital schistosomiasis diagnosis in endemic areas is the microscopic quantification of *S. haematobium* eggs in urine using polycarbonate filters [7, 11]. However, after praziquantel treatment and repeated preventive chemotherapy, macro- and microhaematuria decrease, as well as intensities of infection and the overall prevalence [12–15]. Hence, in areas that have achieved morbidity control (prevalence of heavy-intensity infection < 5% across sentinel sites) and move towards elimination of urogenital schistosomiasis as public health problem (prevalence of heavy-intensity infections < 1% in all sentinel sites) and finally interruption of transmission (reduction of incidence of infection to zero) according to WHO thresholds [4], macro- and microhaematuria and the number of eggs excreted in urine will be extremely low and eventually zero. These light intensity infections impose a challenge for accurate diagnosis.

A large dataset with *S. haematobium* diagnostic results was derived from a 5-year cluster randomized trial assessing different interventions against urogenital schistosomiasis in Zanzibar funded by the Schistosomiasis Consortium for Operational Research and Evaluation (SCORE) [16]. Zanzibar is one of the first areas in sub-Saharan Africa that is targeted for urogenital schistosomiasis elimination as public health problem and interruption of transmission. Using these data, we aimed to assess whether the sensitivity of microhaematuria testing using reagent strips increases with increasing egg counts measured with the standard urine filtration method. In addition, we aimed to determine the diagnostic test sensitivity at different light intensity infection sub-groups.

## Methods

### Study area

The Zanzibar islands, Unguja and Pemba, are part of the United Republic of Tanzania. The population size is estimated at 1.3 million. Historically, urogenital schistosomiasis has been a considerable public health problem on both islands [17–19]. Over the past decades, regular mass drug administration (MDA) with praziquantel, improved access to safe water, better socio-economic conditions and likely also climatic changes have lowered the disease prevalence and reduced morbidity [20, 21]. Efforts to eliminate urogenital schistosomiasis as public health problem on Pemba and to interrupt transmission on Unguja started in 2011 by the Zanzibar Elimination of Schistosomiasis Transmission (ZEST) alliance [16, 22]. These efforts were fostered by a three-arm multi-year cluster randomized trial implemented from 2011 to 2017 to assess the effect of biannual MDA, snail control and behaviour change interventions [16]. To date, with the exception of some areas where transmission is still considerably high [21], the prevalence of *S. haematobium* infections is well below 10% and infection intensities are light in most administrative areas (shehias).

### Sample size calculation

The sample size calculations for the cluster randomized trial and annual cross-sectional surveys in schools and communities are provided elsewhere [16]. The results of all individuals with a complete urine examination, by urine filtration and reagent strip methods, in 2013, 2014, 2015 and 2016, were included into the analyses presented here.

### Field procedures

The cross-sectional surveys in schools and communities were conducted annually, in both Unguja and Pemba, between February and June in 2013, 2014, 2015 and 2016. Children aged 9–12 years attending the study primary schools and adults aged 20–55 years living in the study communities were included. In each primary school, the headmaster and teachers were informed about the aims of the study and in each community the community leader was consulted. The participant selection procedure in schools and communities has been described elsewhere in detail [16]. In brief, in public primary schools, classes of grades 3 and 4 were visited by the field teams of the NTD Programme and the Public Health Laboratory-Ivo de Carneri (PHL-IdC) on Unguja and Pemba, respectively. The purpose of the study was explained in lay terms to the children. The name, age, sex, and additional demographic information of the selected children were recorded. The children registered for participation received an information sheet and a consent form to bring to their parents. The following day, each child returning the consent form signed by its parent or legal guardian received a urine collection container and was asked to fill the container with its own urine (urine collection occurred between 10:00 and 12:00 hours) and to give the filled container to the field team. In each shehia, households were randomly selected and an adult household member, present at the time, was invited to participate in the study [16]. After consenting and replying to a brief questionnaire concerning demographic characteristics, the adult received a container for collection of his/her own urine. All urine samples from adults were collected between 10:00 and 14:00 hours. The urine samples collected in Unguja were examined in the laboratory of the NTD Programme of the Ministry of Health in Zanzibar Town, Unguja. The urine samples collected in Pemba were examined in the PHL-IdC in Chake Chake, Pemba.

### Laboratory procedures

On the day of collection, all urine samples (i.e. one single sample per person) were examined by trained laboratory technicians for macrohaematuria using a colour chart and for microhaematuria using reagent strips (Hemastix; Siemens Healthcare Diagnostics GmbH, Eschborn, Germany). Macrohaematuria was graded with numbers from 1 to 6 from transparent to dark red urine using a pretested colour chart [23, 24]. Microhaematuria in urine was coded semi-quantitatively according to the Hemastix manufacturer’s instructions (negative; trace; +; ++; and +++). Additionally, all urine samples of sufficient quantity were rigorously shaken and 10 ml of each sample pressed through a polycarbonate filter with a pore size of 20 μm (Sterlitech, Kent, WA, USA) using a standard 10 ml plastic syringe. All urine filters were put on a microscope slide, covered with a piece of hydrophilic cellophane soaked in glycerol solution, and examined by trained laboratory technicians under the microscope using some drops of Lugol’s iodine to stain *S. haematobium* eggs after cellophane coverage. The presence and number of *S. haematobium* eggs was recorded. After microscopy, the slides were stored at room temperature for a potential second reading for quality control. Quality control was performed on 10% of the stored urine filtration slides several months after the initial reading. For the selection of quality control urine filtration (QCUF) slides, 10% of the slides of each technician were included, prioritizing slides from microhaematuria-positive urine samples and adding computer randomized microhaematuria-negative slides until the number representing 10% of the total number of slides read by the technician was reached. The QCUF slides were read by trained external microscopists who were blinded to the reagent strip and initial urine filtration results.

### Data management and analysis

The macrohaematuria, microhaematuria, urine filtration, and QCUF results were recorded on paper laboratory forms and subsequently entered into a Microsoft Excel 2010 electronic database (Microsoft Corporation 2010) and cleaned. Data were analysed using STATA version 14.0 (StataCorp., College Station, TX, USA). Only data from urine samples with complete examination (i.e. microhaematuria, microhaematuria and urine filtration result available) and from children aged 9-12 years or adults aged 20-55 years were included in the analyses.

Microhaematuria-positive was defined as a urine sample that had a trace or positive reagent strip colour reaction. *S. haematobium*-positive was defined as a urine filtration slide that contained at least one *S. haematobium* egg. The WHO differentiates *S. haematobium* infections into light (1–49 eggs per 10 ml urine) and heavy (≥ 50 eggs per 10 ml urine) intensity [25]. In our study we further stratified egg counts into the following sub-classes: “negative” (0 eggs/10 ml), “ultra-light” (1–5 eggs/10 ml), “very light” (6–10 eggs/10 ml), “light” (11–49 eggs/10 ml) and “heavy” (≥ 50 eggs/10 ml) infections. Association between *S. haematobium* infection (binary outcome variable or categorical explanatory variable) and microhaematuria (binary outcome variable or categorical explanatory variable) was assessed by multivariable logistic regression analyses, adjusted by sex (binary variable), age (continuous variable), study year (categorical variable) and school or shehia as a sampling unit (categorical variable) and expressed as odds ratios (OR) plus 95% confidence intervals (95% CI).

For both children and adults separately, the sensitivity and specificity of the reagent strip method was calculated overall and stratified by the egg count thresholds chosen for ultra-light, very light, light, and heavy infections as described above. The original urine filtration was considered as the diagnostic reference test. The sensitivity of a reagent strip result was calculated as the proportion of positives that were correctly identified when compared to the reference test. The specificity of a reagent strip result was calculated as the proportion of negatives that were correctly identified when compared to the reference test. We used 95% CIs to indicate the contrast between groups. In addition, we used logistic regression to assess whether the sensitivity of the reagent strip method is increasing with increasing egg counts determined by the urine filtration method. For this purpose we used the decimal logarithm of the egg counts of the filtration-positive samples as predictor. The graphical representation of the predicted values is shown in Additional file 1.

The agreement between the positive and negative readings of the original urine filtration *versus* the QCUF reading was determined using kappa (κ)-statistics. The κ-statistics were interpreted as follows: < 0.00 indicating no agreement; 0.00–0.20 indicating slight agreement; 0.21–0.40 indicating fair agreement; 0.41–0.60 indicating moderate agreement; 0.61–0.80 indicating substantial agreement; 0.81–0.99 indicating almost perfect agreement; and 1.00 indicating perfect agreement [26].

## Results

### Study participation and operational results

**Fig. 1:**
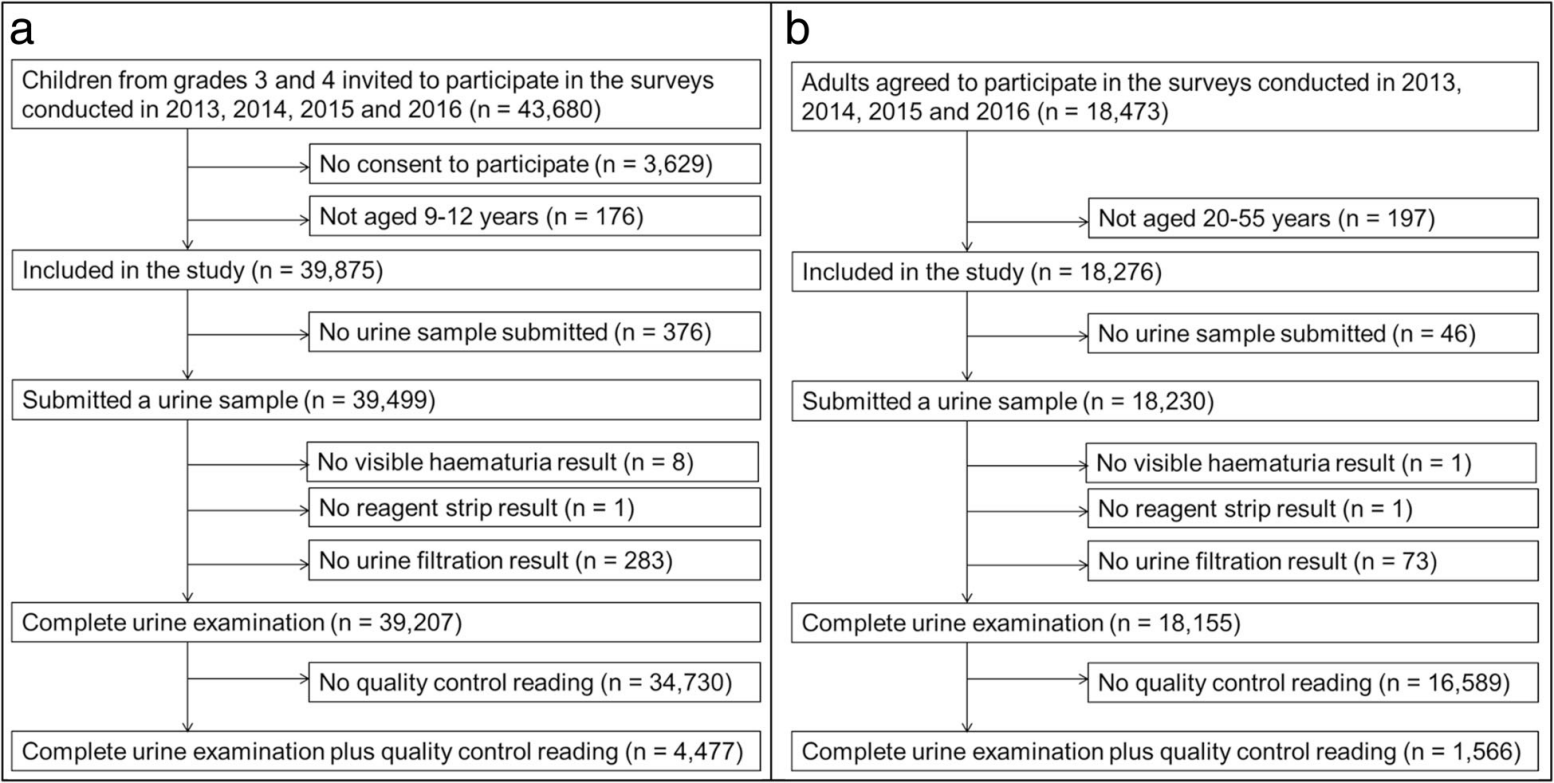
Flowchart detailing study participation and urine sampling procedures. **a** Children sampled in public primary schools. **b** Adults sampled in communities in Unguja and Pemba islands, United Republic of Tanzania

As shown in Fig. 1a, a total of 43,680 children were invited to participate in the cross-sectional surveys conducted in 2013, 2014, 2015 and 2016. Among them, 39,875 were aged 9–12 years and submitted a signed form from their parents consenting to their participation. Complete urine sample examinations including results on macrohaematuria, microhaematuria and *S. haematobium* egg counts were available for 39,207 children. Among them, 20,680 (52.7%) were girls and 18,527 (47.3%) were boys. A QCUF reading was performed for 4477 slides.

Figure 1b indicates that a total of 18,473 adults participated in the study. Among them, 18,276 were aged 20–55 years and included in the study. Complete urine sample examinations were available for 18,155 adults. Among them, 10,573 (58.2%) were female and 7582 (41.8%) were male. A QCUF reading was performed for 1566 slides.

### Association between infection intensity and haematuria in children

As shown in Table 1, among the 39,207 urine filtration slides examined for *S. haematobium* infection in children, 2130 (5.4%) were found to be egg-positive. Among the *S. haematobium*-positive slides, ultra-light infections with 1–5 eggs/10 ml urine were most common (34.7%). Very light infections with 6–10 eggs/10 ml were found in 13.3%, light infections with 11–49 eggs/10 ml were found in 30.3%, and heavy infections with ≥ 50 eggs/10 ml urine were found in 21.6% of the *S. haematobium*-positive slides. Among all urine samples examined, 1.2% were identified with heavy infection intensities.

**Table 1:** Multivariate frequency distribution of *S. haematobium* infection and egg counts and haematuria presence and grading

Urine filtration original reading			Microhaematuria			Microhaematuria grading				Macrohaematuria grading					
	n	%	*n* negative	*n* positive including trace	% positive	Trace	+	++	+++	1	2	3	4	5	6
Total children examined	39,207		36,675	2532	6.5										
*S. haematobium* egg-negative	37,077	94.6	36,070	1007	2.7	430	264	205	108	19,070	14,265	3508	223	11	0
*S. haematobium* egg-positive	2130	5.4	605	1525	71.6	292	290	468	475	801	1030	279	18	2	0
*S. haematobium* egg counts															
1–5 eggs	740	34.7	369	371	50.1	102	91	116	62	332	216	89	30	0	0
6–10 eggs	284	13.3	85	199	70.1	52	41	62	44	117	137	28	2	0	0
11–49 eggs	646	30.3	119	527	81.6	103	113	167	144	234	325	81	6	0	0
50+ eggs	460	21.6	32	428	93.0	35	45	123	225	118	252	81	7	2	0
Total adults examined	18,155		16,131	2024	11.1										
*S. haematobium* egg-negative	17,673	97.3	15,985	1688	9.6	541	475	383	289	7275	7972	2250	170	5	1
*S. haematobium* egg-positive	482	2.7	146	336	69.7	65	62	99	110	157	245	67	11	2	0
*S. haematobium* egg counts															
1–5 eggs	225	46.7	93	132	58.7	28	40	29	35	80	109	31	5	0	0
6–10 eggs	64	13.3	18	46	71.9	11	5	19	11	25	27	9	2	1	0
11–49 eggs	124	25.7	27	97	78.2	17	14	32	34	38	71	13	1	1	0
50+ eggs	69	14.3	8	61	88.4	9	3	19	30	14	38	14	3	0	0

Table 1 indicates that the majority of urines were lightly coloured and only very few children and adults had visible haematuria. A total of 2532 (6.5%) urine samples from children were microhaematuria-positive. Among the 2130 *S. haematobium* egg-positive urine samples, 605 (71.6%) were microhaematuria-positive and among the 37,077 *S. haematobium* egg-negative urine samples, 1007 (2.7%) were microhaematuria-positive.

**Fig. 2:**
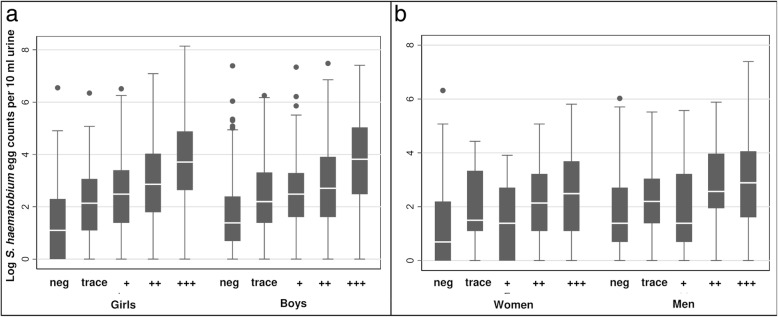
Boxplots of *S. haematobium* log egg counts of egg-positive urine samples from Zanzibar, United Republic of Tanzania, stratified by microhaematuria grading, sex and age category. **a** log *S. haematobium* egg-counts by microhaematuria grading for female and male children. **b** log *S. haematobium* egg-counts by microhaematuria grading for female and male adults. Correlation between positive egg counts and microhaematuria grading (data pooled over both sexes): Spearman’s rho in children= 0.65, P < 0.001 (n = 39,207), Spearman’s rho in adults = 0.32; P < 0.001 (n = 18,155). In contrast to the boxplots, the correlation coefficients were calculated using egg counts from positive and negative urine samples

Compared with *S. haematobium* egg-negative children, egg-positive children had significantly higher odds to present with microhaematuria (OR: 85.7; 95% CI: 74.9–98.1). The odds increased with increasing egg counts and were highest for heavy (OR: 604.2; 95% CI: 414.5–880.8), followed by light (OR: 208.7; 95% CI: 166.0–265.5), very light (OR: 96.1; 95% CI: 72.8–126.9), and ultra-light (OR: 45.0; 95% CI: 37.6–53.9) infections. Boys had higher odds to be *S. haematobium*-positive (OR: 1.9; 95% CI: 1.7–2.2) but lower odds to be microhaematuria-positive (OR: 0.9; 95% CI: 0.7–0.9) than girls. More details about the associations between *S. haematobium* infection intensity and microhaematuria are presented in Table 2 and Fig. 2a.

**Table 2:** Association between *S. haematobium* egg counts and microhaematuria

Variable type	Variable name	*n*	OR	95% CI
Children aged 9–12 years		39,207		
Outcome	*S. haematobium* egg-positive			
Explanatory	Microhaematuria-positive		86.6	75.6–99.1
	Trace		42.8	35.0–52.3
	+		66.8	53.4–83.2
	++		129.6	104.2–161.2
	+++		188.8	146.5–243.3
	Boys		1.9	1.67–2.17
Outcome	Microhaematuria-positive			
Explanatory	*S. haematobium* egg-positive		85.7	74.9–98.1
	1-5 eggs		45	37.6–53.9
	6-10 eggs		96.1	72.8–126.9
	11-49 eggs		208.7	166.0–262.5
	50+ eggs		604.2	414.5–880.8
	Boys		0.83	0.74–0.93
Adults aged 20–55 years		18,155		
Outcome	*S. haematobium* egg-positive			
Explanatory	Microhaematuria-positive		29.6	23.6–37.2
	Trace		19.3	13.7–27.1
	+		21.6	15.3–27.1
	++		34.6	25.3–47.3
	+++		50.2	36.4–69.3
	Men		2.5	2.0–3.1
Outcome	Microhaematuria-positive			
Explanatory	*S. haematobium* egg-positive		29.5	23.6–36.8
	1–5 eggs		19.9	15.0–26.3
	6–10 eggs		42.3	24.2–74.0
	11–49 eggs		50.6	32.0–79.9
	50+ eggs		129.1	60.6–274.8
	Men		0.5	0.44–0.55

*Abbreviations*: *OR* odds ratio; *95% CI* 95% confidence interval

### Association between infection intensity and haematuria in adults

As shown in Table 1, among the 18,155 urine filtration slides examined for *S. haematobium* infection in adults, 482 (2.7%) were egg positive. Ultra-light infections were most common (46.7%), followed by light (25.7%), heavy (14.3%) and very light (13.3%) infections. Among all examined urine samples, 0.4% were identified with heavy infection intensities. Among the *S. haematobium* egg-positive samples, 336 (69.7%) were microhaematuria-positive and among the egg-negative urine samples, 1688 (9.6%) were microhaematuria-positive.

Adults had higher odds to be microhaematuria-positive if *S. haematobium* eggs were found in their urine (OR: 29.5; 95% CI: 23.6–36.8). The odds increased with increasing egg counts (Table 2). They were highest for heavy (OR: 129.1; 95% CI: 60.6–274.8), followed by light (OR: 50.6; 95% CI: 32.0–79.9), very light (OR: 42.3; 95% CI: 24.2–74.0), and ultra-light (OR: 19.9; 95% CI: 15.0–26.3) infections. Men had higher odds to be *S. haematobium*-positive (OR: 2.5; 95% CI: 2.0–3.1) but lower odds to be microhaematuria-positive (OR: 0.50; 95% CI: 0.4–0.6) than women.

### Specificity and sensitivity of the reagent strip method

As shown in Table 3, in children, the specificity of the reagent strip method was 97.3% (97.1–97.4%). The overall sensitivity was 71.6% (95% CI: 69.6–73.5%). When considering intensity subgroups, the sensitivity was lowest for ultra-light infections (50.1%; 95% CI: 46.5–53.8%), followed by very light (70.1%; 95% CI: 64.4–75.3%), light (81.6%; 95% CI: 78.4–84.5%), and heavy (93.0%; 95% CI: 90.3–95.2%) infections.

**Table 3:** Sensitivity and specificity of the reagent strip method for *S. haematobium* diagnosis in children when urine filtration results are considered as reference test

Reference test: urine filtration		Microhaematuria		
All individuals included		Positive	Negative	Total
	Positive	1525	605	2130
	Negative	1007	36,070	37,077
	Total	2532	36,675	39,207
Sensitivity of reagent strips		71.6% (69.6–73.5%)		
Specificity of reagent strips		97.3% (97.1–97.4%)		
Subgroup: 1–5 eggs/10 ml		Positive	Negative	Total
	Positive	371	369	740
	Negative	1007	36,070	37,077
	Total	1378	36,439	37,817
Sensitivity of reagent strips		50.1% (46.5–53.8%)		
Subgroup: 6–10 eggs/10 ml		Positive	Negative	Total
	Positive	199	85	284
	Negative	1007	36,070	37,077
	Total	1206	36,155	37,361
Sensitivity of reagent strips		70.1% (64.4–75.3%)		
Subgroup: 11–49 eggs/10 ml		Positive	Negative	Total
	Positive	527	119	646
	Negative	1007	36,070	37,077
	Total	1534	36,189	37,723
Sensitivity of reagent strips		81.6% (78.4–84.5%)		
Subgroup: 50+ eggs/10 m		Positive	Negative	Total
	Positive	428	32	460
	Negative	1007	36,070	37,077
	Total	1435	36,102	37,537
Sensitivity of reagent strips		93.0% (90.3–95.2%)		

As indicated in Table 4 among adults, the specificity of the reagent strip method was 90.4% (95% CI: 90.0–90.9%). The overall sensitivity was 69.7% (95% CI: 65.4–73.8%). Stratified by intensity, the sensitivity was 58.7% (95% CI: 51.9–65.2%) for ultra-light, 71.9% (95% CI: 59.2–82.4%) for very light, 77.9% (95% CI: 69.5–84.9%) for light and 88.7% (95% CI: 79.0–95.0%) for heavy infections.

**Table 4:** Sensitivity and specificity of the reagent strip method for *S. haematobium* diagnosis in adults when urine filtration results are considered as reference test

Reference test: urine filtration		Microhaematuria		
All individuals included		Positive	Negative	Total
	Positive	336	146	482
	Negative	1688	15,985	17,673
	Total	2024	16,131	18,155
Sensitivity of reagent strips		69.7% (65.4–73.8%)		
Specificity of reagent strips		90.4% (90.0–90.9%)		
Subgroup: 1–5 eggs/10 ml		Positive	Negative	Total
	Positive	132	93	225
	Negative	1688	15,985	17,673
	Total	1820	16,078	17,898
Sensitivity of reagent strips		58.7% (51.9–65.2%)		
Subgroup: 6–10 eggs/10 ml		Positive	Negative	Total
	Positive	46	18	64
	Negative	1688	15,985	17,673
	Total	1734	16,003	17,737
Sensitivity of reagent strips		71.9% (59.2–82.4%)		
Subgroup: 11–49 eggs/10 ml		Positive	Negative	Total
	Positive	95	27	122
	Negative	1688	15,985	17,673
	Total	1783	16,012	17,795
Sensitivity of reagent strips		77.9% (69.5–84.9%)		
Subgroup: 50+ eggs/10 ml		Positive	Negative	Total
	Positive	63	8	71
	Negative	1688	15,985	17,673
	Total	1751	15,993	17,744
Sensitivity		88.7% (79.0–95.0%)		

Additional file 1 shows that the sensitivity of the reagent strip method increased significantly for each unit log_10_ egg count per 10 ml urine in children (OR: 4.7; 95% CI: 4.0–5.7, *P* < 0.0001) and adults (OR: 2.6; 95% CI: 1.9–3.7, *P* < 0.0001). The difference of the test sensitivity between children and adults (*P* = 0.001) as well as the population-egg-count interaction (*P* = 0.002) were statistically significant.

### Urine filter microscopy

Table 5 shows that among the 4477 urine filtration slides from children that were subjected to QCUF, 3087 slides were negative and 1096 slides were recorded as egg-positive in both the original and the QCUF reading. The kappa-agreement was almost perfect (κ = 0.84). However, 163 slides were only positive in the original and 131 slides were only positive in the QCUF reading. As presented in Table 5, among the 294 slides that were only positive in one or the other microscopy reading, 93 slides had an egg count of 1 (31.6%). The vast majority of the discrepant slides had egg counts between 1 and 5 (76.9%), followed by egg counts between 6 and 10 (12.2%), egg counts between 11 and 49 (7.5%), and 50 and above (3.4%).

**Table 5:** *Schistosoma haematobium* egg counts on discrepant slides when examined by original or quality control urine filtration (QCUF) microscopy

Microscopic examination		*n*	%
Children	Slides with original and QCUF reading	4477	
	Slides with positive results in both readings	1096	
	Slides with negative results in both readings	3087	
	Slides with discrepant results (one reading positive, one reading negative)	294	
	*S. haematobium* egg counts on discrepant slides		
	1 egg	93	31.6
	2 eggs	57	19.4
	3 eggs	39	13.3
	4 eggs	23	7.8
	5 eggs	14	4.8
	1–5 eggs	226	76.9
	6–10 eggs	36	12.2
	11–49 eggs	22	7.5
	50+ eggs	10	3.4
Adults	Slides with original and QCUF reading	1566	
	Slides with positive results in both readings	199	
	Slides with negative results in both readings	1292	
	Slides with discrepant results (one reading positive, one reading negative)	75	
	*S. haematobium* egg counts on discrepant slides		
	1 egg	34	45.3
	2 eggs	12	16.0
	3 eggs	6	8.0
	4 eggs	7	9.3
	5 eggs	4	5.3
	1–5 eggs	63	84.0
	6–10 eggs	10	13.3
	11–50 eggs	1	1.3
	51+ eggs	1	1.3

A total of 1566 urine filtration slides from adults were subjected to QCUF. Among them, 1292 were negative and 199 were recorded as egg-positive in both the original and the QCUF reading. Hence, the kappa-agreement was almost perfect (κ = 0.81). However, 35 slides were only positive in the original reading and 40 slides were only positive in the QCUF reading. For adults, among the 75 slides that had discrepant results, almost half (45.3%) had an egg count of 1. The majority of the discrepant slides, had egg counts between 1 and 5 (84.0%), followed by egg counts between 6 and 10 (13.3%), egg counts between 11 and 49 (1.3%), and 50 and above (1.3%).

## Discussion

Reagent strips and urine filtration are basic standard diagnostic methods to detect urogenital schistosomiasis [7, 27–29]. Their sensitivity is, however, reduced in low prevalence settings, treated populations, or subgroups with light intensity infections [27, 30, 31]. Moving towards the goal of interruption of *Schistosoma* transmission, it is important to assess at what level of egg counts the diagnostic methods start to lose their accuracy. Here, we evaluated the performance of urine filtration readings and microhaematuria assessment for diagnosing *S. haematobium* infections in several thousand urine samples from children and adults living in Zanzibar, an area targeted for interruption of transmission of urogenital schistosomiasis.

Our study clearly showed that both reagent strip and urine filtration method have a particularly low diagnostic accuracy for the detection of ultra-light infections with egg counts of 1–5 eggs per 10 ml of urine. In line with a recent meta-analysis, indicating sensitivities of reagent strips of 65% for light intensity infection and 72% for post-treatment groups [30], the overall sensitivity to detect *S. haematobium* infections with reagent strips in children and adults in our study was 71.6% and 69.7%, respectively. However, the sensitivity of reagent strips to detect ultra-light infections was considerably lower (50.1% in children and 58.7% in adults). Comparing the results of urine filtration microscopy, we found that the overall agreement when slides were read twice was almost perfect. However, false-negative diagnosis by one of the two readings occurred and particularly when egg counts were between 1 and 5 eggs per 10 ml urine.

In Zanzibar, only a small portion of the samples examined by urine filtration were *S. haematobium* egg-positive (5.4% of children and 2.7% of adults) and more than a third of these egg-positive slides showed ultra-light infections with 1–5 eggs per 10 ml urine (34.7% of children and 46.7% of adults). Only about half of these ultra-lightly infected individuals (50.1% of children and 58.7% of adults) presented with detectable microhaematuria. Our results highlight that a large proportion of individuals living in elimination settings such as Zanzibar that are targeted by regular interventions harbour ultra-light infections. These cases may be missed when reagent strips or single urine filtration readings are applied as diagnostic approach. Hence, in settings where interruption of transmission is the goal and already the excretion of a single egg into a water body with intermediate host snails can result in resurgence of transmission and (re-)infection of a whole community, more sensitive diagnostic methods are needed to identify and subsequently treat infected individuals. These next-generation diagnostic tools should be able to reliably detect infections with ≤ 5 eggs per 10 ml urine.

In line with other studies, the performance of both reagent strips and urine filtration improved significantly when egg counts increased [12, 18, 27]. The odds of urine samples being microhaematuria-positive increased significantly from ultra-light to very light to light to heavy infection egg outputs. Also, the number of false-negative or false-positive urine filtration slide readings decreased considerably with increasing egg counts. Only very few slides were wrong-negative or wrong-positive at counts of ≥ 50 eggs per 10 ml urine, errors that might be attributed to wrong labelling.

Hence, urine filtration and reagent strip methods are valid means to detect *S. haematobium* infections in epidemiological surveys and outpatient centres in areas where infection intensities are reasonably high [30, 32]. In areas identified to have high prevalences and infection intensities, preventive chemotherapy without individual diagnosis will be the key intervention to control morbidity [7]. Urine filtration and reagent strips might also be suitable tools for monitoring progress in areas where preventive chemotherapy plus complementary interventions are used to achieve elimination as a public health problem. However, only urine filtration allows classification of *S. haematobium* infections into light and heavy intensities as defined by the WHO [25]. Where interruption of transmission is the goal, novel intervention strategies need to be considered and tested. The sensitive and specific identification of infected individuals excreting *S. haematobium* eggs, including ultra-lightly infected individuals, will gain importance. Hence, next-generation diagnostic tools that are reliably working below a level of 5 eggs per 10 ml urine are urgently needed.

A first step into improved diagnosis of *S. haematobium* infections in population groups excreting low numbers of eggs was done with the development and evaluation of the up-converting phosphor-lateral flow circulating anodic antigen (UCP-CAA) assay and the detection of parasite specific DNA *Dra*1 fragments in urine using PCR-based methods, respectively [29, 31, 33–35]. However, these tests need a considerable amount of equipment, material and training of technicians and will hence mostly be of use in well-equipped central laboratories. For monitoring and surveillance at the peripheral level, e.g. in local schools and health facilities, a simple rapid diagnostic test with high sensitivity such as the point-of-care circulating cathodic antigen test available for *S. mansoni* [36, 37] is needed also for *S. haematobium* diagnosis. Such a sensitive rapid diagnostic test would facilitate focal screen-and-treat and other tailored surveillance-response scenarios that could become part of a strategy for interrupting *S. haematobium* transmission.

A clear limitation of our study is that no third and highly sensitive diagnostic method such as the UCP-CAA or PCR was used to validate results derived with the urine filtration and reagent strip methods. Also the collection of multiple urine samples from the same individuals would have allowed to assess more thoroughly the relationship between low egg intensity and macro- or microhaematuria while accounting for variation between individuals as well as changes in egg excretion during the day. Nevertheless, the analysis of our large dataset allows for the following conclusions and considerations.

## Conclusions

We found that ultra-light *S. haematobium* infections are most common in Zanzibar and impose a major challenge for accurate diagnosis using basic parasitological methods. Next-generation diagnostic tools to be used in settings where interruption of transmission is the goal should reliably detect infections ≤ 5 eggs per 10 ml urine. These new tests should not only be highly sensitive, but also rapid and easy to apply so that they can be used for surveillance at the central and peripheral level, triggering an effective and focused intervention response.

## Additional file

Additional file 1: Predicted sensitivity of reagent strips by infection intensity estimated by urine filtration. The sensitivity of the reagent strip method to detect microhaematuria increased significantly for each unit log_10_ egg count per 10 ml urine in children (odds ratio, OR: 4.7; 95% confidence interval, CI: 4.0–5.7, *P* < 0.0001) and adults (OR: 2.6; 95% CI: 1.9–3.7, *P* < 0.0001). The difference of the test sensitivity between children and adults (*P* = 0.001) as well as the population-egg-count interaction (*P* = 0.002) were statistically significant. (PDF 32 kb)
