# Correction to: Urogenital schistosomiasis elimination in Zanzibar: accuracy of urine filtration and haematuria reagent strips for diagnosing light intensity *Schistosoma haematobium* infections

**DOI:** 10.1186/s13071-019-3397-8

**Published:** 2019-03-28

**Authors:** Stefanie Knopp, Shaali M. Ame, Jan Hattendorf, Said M. Ali, Iddi S. Khamis, Faki Bakar, Mwanaidi A. Khamis, Bobbie Person, Fatma Kabole, David Rollinson

**Affiliations:** 1 Swiss Tropical and Public Health Institute, Socinstrasse 57, 4002 Basel, Switzerland; 2 University of Basel, Petersplatz 1, 4001 Basel, Switzerland; 3 Wolfson Wellcome Biomedical Laboratories, Department of Life Sciences, Natural History Museum, Cromwell Road, London, SW7 5BD UK; 4 Public Health Laboratory Ivo-de Carneri, P.O. Box 122, Chake-Chake, Pemba United Republic of Tanzania; 5 Neglected Diseases Programme, Ministry of Health, P.O. Box 236, Unguja, United Republic of Tanzania; 6 Schistosomiasis Consortium for Operational Research and Evaluation, 145 Coverdell Center, The University of Georgia, 500 D.W. Brooks Drive, Athens, GA 30602 USA

## Correction to: Parasites & Vectors (2018) 11:552 10.1186/s13071-018-3136-6

Following publication of the original article [1], the authors flagged that unfortunately an error had been introduced to the Conclusions section of the article’s Abstract, during production of the article.

The original and wrongly published section reads as (*see the error in bold):

- “We found that many individuals infected with *S. haematobium* in Zanzibar excrete **> 5 eggs** per 10 ml urine (…)”

However, it should read as (*see the correction in bold):

- “We found that many individuals infected with *S. haematobium* in Zanzibar excrete **less than 5 eggs** per 10 ml urine (…)”

The original article has now been updated to correct this error.

The publisher apologizes for the processing error.
