# Supplementary material for: Urogenital schistosomiasis elimination in Zanzibar: accuracy of urine filtration and haematuria reagent strips for diagnosing light intensity Schistosoma haematobium infections
Source: Parasit Vectors. 2018 Oct 23;11:552. doi: 10.1186/s13071-018-3136-6 (PMC6199745; doi:10.1186/s13071-018-3136-6)

Additional file 1

Predicted sensitivity of reagent strips by infection intensity estimated by urine filtration

Method: We used logistic regression to assess whether the sensitivity of the reagent strip method is increasing with increasing egg counts determined by the urine filtration method. For this purpose we used the decimal logarithm of the egg counts of the filtration-positive samples as predictor.

Results: As shown in the figure below, the sensitivity of the reagent strip method to detect microhaematuria increased significantly for each unit log10 egg count per 10 ml urine in children (odds ratio [OR]: 4.7, 95% confidence interval (CI): 4.0 - 5.7, P < 0.0001) and adults (OR: 2.6, 95% CI: 1.9 - 3.7, P < 0.0001). The difference between children and adults was statistically significant (P = 0.001) as well as the population-egg-count interaction (P = 0.002).

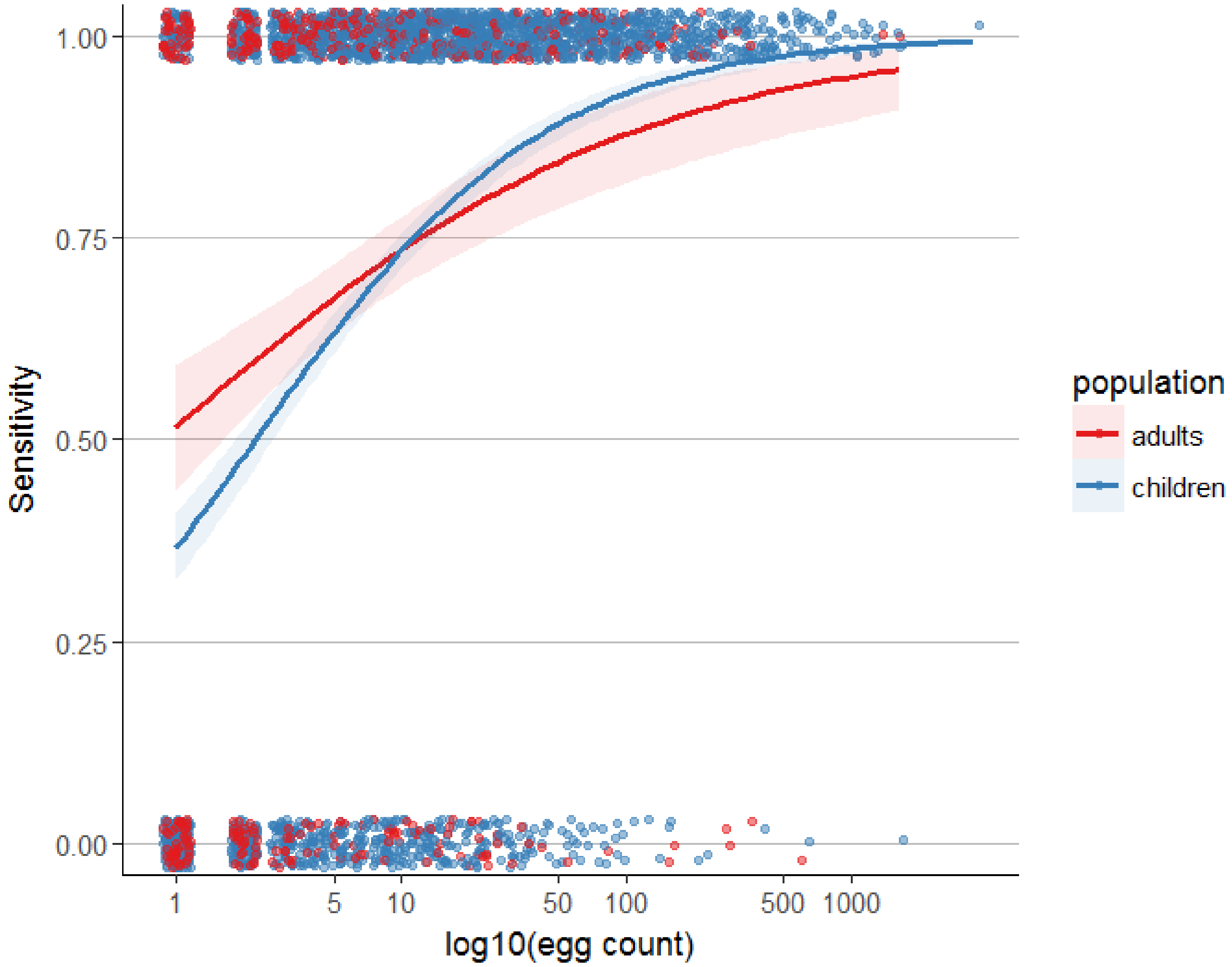

Supplement: Supplementary file 1 — Predicted sensitivity of reagent strips by infection intensity estimated by urine filtration. The sensitivity of the reagent strip method to detect microhaematuria increased significantly for each unit log10 egg count per 10 ml urine in children (odds ratio, OR: 4.7; 95% confidence interval, CI: 4.0–5.7, P < 0.0001) and adults (OR: 2.6; 95% CI: 1.9–3.7, P < 0.0001). The difference of the test sensitivity between children and adults (P = 0.001) as well as the population-egg-count interaction (P = 0.002) were statistically significant. (PDF 32 kb) [file 13071_2018_3136_MOESM1_ESM.pdf]
